# Intra-specific variability and biological relevance of P3N-PIPO protein length in potyviruses

**DOI:** 10.1186/1471-2148-13-249

**Published:** 2013-11-13

**Authors:** Julia Hillung, Santiago F Elena, José M Cuevas

**Affiliations:** 1 Instituto de Biología Molecular y Celular de Plantas, Consejo Superior de Investigaciones Científicas-Universidad Politécnica de Valencia, València 46022, Spain; 2 The Santa Fe Institute, 87501, Santa Fe, NM, USA; 3 Present address: Institut Cavanilles de Biodiversitat i Biologia Evolutiva, Universitat de València, València 46980, Spain

**Keywords:** Bayesian phylogenetic methods, Host-range determinants, Molecular evolution, *Potyvirus*, Virus evolution, Virus fitness components

## Abstract

**Background:**

*Pipo* was recently described as a new ORF encoded within the genome of the *Potyviridae* family members (PNAS 105:5897–5902, 2008). It is embedded within the *P3* cistron and is translated in the +2 reading frame relative to the potyviral long ORF as the P3N-PIPO fusion protein. In this work, we first collected *pipo* nucleotide sequences available for different isolates of 48 *Potyvirus* species. Second, to determine the biological implications of variation in *pipo* length, we measured infectivity, viral accumulation, cell-to-cell and systemic movements for two *Turnip mosaic virus* (TuMV) variants with *pipo* alleles of different length in three different susceptible host species, and tested for differences between the two variants.

**Results:**

In addition to inter-specific variation, there was high variation in the length of the PIPO protein among isolates within species (ranging from 1 to 89 amino acids). Furthermore, selection analyses on the *P3* cistron did not account for the existence of stop codons in the *pipo* ORF, but showed that positive selection was significant in the overlapping region for *Potato virus Y* (PVY) and TuMV. In some cases, variability in length was associated with host species, geographic provenance and/or other strain features. We found significant empirical differences among the phenotypes associated with TuMV *pipo* alleles, though the magnitude and sign of the effects were host-dependent.

**Conclusions:**

The combination of computational molecular evolution analyses and experiments stemming from these analyses provide clues about the selective pressures acting upon the different-length *pipo* alleles and show that variation in length may be maintained by host-driven selection.

## Background

The *Potyviridae* are one of the largest plant virus families, and many of its members represent major agricultural threats [1,2]. This family belongs to the picorna-like virus superfamily, for which overlapping ORFs have been described only in a few cases [3-5]. Until very recently, the genomic RNA of the *Potyvirus* genus, the largest genus within the *Potyviridae*, was thought to contain a single functional ORF encoding a polyprotein that is cleaved into 10 mature proteins (P1, HC-Pro, P3, 6 K1, CI, 6 K2, VPg, NIa-Pro, NIb, and CP) by three viral proteases: P1, HC-Pro and NIa-Pro [6]. However, Chung *et al*. [7] provided evidence of the existence of a new ORF in the reference sequences of 48 potyvirus species; and named it *pipo* (Pretty Interesting *Potyviridae* ORF). The *pipo* ORF is embedded within the *P3* cistron and is translated in the +2 reading frame relative to the long ORF. *Pipo* is expressed as a fusion product with the N-terminal portion of P3 protein (ca. 25 kDa), P3N-PIPO, via ribosomal frameshifting or transcriptional slippage at a highly conserved motif at the 5′ end of *pipo* ORF [7]. Its length is quite variable among the different potyvirus species, ranging from 60 to 115 codons (inter-specific length variation) [7]. Wei *et al.*[8] and Wen & Hajimorad [9] have shown that P3N-PIPO plays a central role as a movement protein. This function is dependent on the interaction between P3N-PIPO and the host hydrophilic cation-binding protein PCaP1; and a knockout of the *PCaP1* gene in *Arabidopsis thaliana* results in a severe reduction of TuMV accumulation and in milder symptoms [10]. Yeast two-hybrid experiments have shown that P3N-PIPO can interact with the small and large subunits of RubisCO, thereby potentially contributing to the development of symptoms [11]. Very recently, P3N-PIPO has been shown to be a virulence determinant that allows infection of plants carrying recessive resistance alleles [12]. However, the effects of genetic variation in *pipo* – in particular protein length – on interactions with host factors and the phenotypic consequences thereof are unknown.

Recently, during a computational survey of PVY variability [13], we made the fortuitous observation that *pipo* length was variable among isolates, with most isolates encoding for PIPO polypeptides of 73–76 amino acids long and one isolate encoding for a much shorter polypeptide of only 62 amino acids. These findings motivate the present study, which is organized in two parts. In the first part, we extended the *in silico* analysis of intra-specific variation in *pipo* length to the 48 potyvirus species used in the seminal study of Chung *et al*. [7]. We first identified those viruses with the largest numbers of isolates and alternative stop codons. For these viruses, we explored whether alternative stop codons may be under selection and whether differences in host species, geographic origin and/or other biological properties of the isolates may create different selective pressures that explain the prevalence of the different alleles in natural populations. The second part of the study is devoted to validating experimentally some of the previous *in silico* findings using two different length alleles of TuMV *pipo*. The first allele encodes the wild-type protein of 60 amino acids, hereafter referred to as *pipo*^*61*^. The second allele, dubbed *pipo*^*70*^, has the stop codons at positions 61 and 65 removed by site-directed mutagenesis, while retaining the stop codon at position 70. Thus, this variant encodes a longer protein consisting of 69 amino acids, which occurs in natural virus isolates. Both mutations at positions 61 and 65 were synonymous in the *P3* reading frame. Then, we tested for allele-specific differences in infectivity, virus accumulation, and the speed of cell-to-cell and systemic movement in three host species: *Nicotiana benthamiana*, *Brassica rapa* and *A. thaliana*.

## Methods

### Alignment and recombination analysis

All available coding sequences that included the *P3* cistron were retrieved from GenBank (48 virus species). Nucleotide sequences were translated and amino acid sequences were aligned with MUSCLE [14] as implemented in MEGA 5 [15]. Finally, the *pipo* ORF was located and its size was assigned for each isolate according to the position of the first stop codon in the +2 reading frame. Global information for the procedure is provided in Additional file 1: Table S1. For each intra-specific dataset showing differences in *pipo* length, the variables host, geographical origin and strain were retrieved whenever available (Additional file 1: Tables S2-S9). As a previous step to any phylogenetic or selection analysis, we performed recombination-detection analyses for the three largest datasets (*Plum pox virus* (PPV), PVY and TuMV) using RDP3 version 3.42 [16] to remove possible mosaic sequences, a common feature among the potyviruses [17-19]. RDP3 was used with the default configuration, except for the option of linear sequence and of disentangling overlapping signals. Only those breakpoints detected by at least three out of the eight methods implemented in RDP3 were accepted. No recombinant sequences were detected.

### Phylogenetic and selection analyses

To check the strength of selection at the codon level, we estimated the differences in nonsynonymous and synonymous substitutions rates per site, *d*_*N*_*– d*_*S*_, using the fixed-effects likelihood (FEL) and internal branches fixed-effects likelihood (IFEL) [20,21]. These methods are implemented in http://www.datamonkey.org[22].

Phylogenetic analyses were performed using the GTR + *Γ*_4_ + I substitution model in a Bayesian MCMC framework, as implemented in BEAST version 1.7 [23]. Substitution rates were estimated using the relaxed uncorrelated exponential clock model. The MCMC was run for 10^8^ generations to ensure convergence of all parameters. The inspection of posterior distributions and the estimation of the relevant evolutionary parameters were done with TRACER version 1.5 (tree.bio.ed.ac.uk/software/tracer). The first 10% of sampled trees were discarded as burn-in. Statistical significance of parameters was evaluated using the 95% highest probability density (HPD). In order to estimate the maximum clade credibility (MCC) phylogeny, including its posterior probabilities, the posterior set of trees (showing branch lengths in substitutions) obtained in BEAST was used. To do so, TREEANNOTATOR version 1.6.1 (beast.bio.ed.ac.uk) was used with 10% of the trees discarded as burn-in. The reliability of the MCC tree was evaluated using 95% HPD confidence intervals.

The CONTRAST program from PHYLIP version 3.69 (evolution.genetics.washington.edu/phylip.html), was used to detect correlations between *pipo* length and each of several biological factors while accounting for the underlying phylogeny. BATS version 1.0b2 [24] was used to elucidate the influence of host species, geographic origin and viral strain in the population structure. BATS computes the parsimony score *PS*[25], the association index *AI*[26] and the maximum monophyletic clade size *MC*[24] statistics, as well as assessing their significance. The first 10% of sampled trees were discarded as burn-in and 10^4^ randomizations were performed to estimate the null distributions of the three statistics.

### TuMV infectious clone

TuMV variants harboring different-length *pipo* alleles were generated by site directed mutagenesis on a plasmid containing TuMV cDNA (GenBank AF530055.2 with a few point mutations; isolate YC5 from calla lily [27]) tagged with GFP. The clone contains stop codons at position 61, 65 and 70 from *pipo* ORF sequence. The stop codon at position 61 was maintained in the wild-type allele *pipo*^*61*^. To generate the longer *pipo*^*70*^ allele, we used the site-directed mutagenesis primers T3141G-PIPO65-2-S (5′-GGGGCAAGCAATGTGTGAAAAACGTACACTCTAG-3′) and T3141G-PIPO65-2-AS (5′-CTAGAGTGTACGTTTTTCACACATTGCTTGCCCC-3′) to replace stop codons at positions 61 and 65 by the most common codons among all the TuMV isolates, which at the same time only introduced synonymous mutations in the *P3* ORF. All constructs were verified by DNA sequencing. DNA extraction was performed with the Qiagen Plasmid Maxi Kit (Qiagen) following the manufacturer’s instructions.

### Measuring viral accumulation

To compare the accumulation of the TuMV-GFP encoding for different P3N-PIPO proteins, 56 four-week old *N. benthamiana*, 19 three-week old *A. thaliana* and 21 four-week old *B. rapa* plants were inoculated with DNA (approximately half of the plants with each *pipo* allele). Five μg of DNA (15 μL) and 5 μL of inoculation buffer (Carborundum 100 mg/mL, 50 mM potassium phosphate, pH 8.0) were mixed on one leaf per plant and gently rubbed. Inoculated plants were maintained in a growth chamber at cycles of 16 h of light at 25°C followed by 8 h of darkness at 22°C. In addition, control plants were inoculated with buffer and maintained in the same conditions.

GFP fluorescence was observed with a Leica MZ16F stereomicroscope, using a 0.5× objective lens, and GFP filters (Leica). Infected plants were randomly selected and harvested at different hours post-inoculation (hpi). Plants were ground into fine powder in a mortar with liquid N_2_, divided into aliquots and stored at -80°C. RNA extraction from 100 mg tissue per plant was performed using InviTrap® Spin Plant RNA Mini Kit (Invitek) following the manufacturer’s instructions. The concentration of total plant RNA extracts was adjusted to 50 ng/μL for each sample. Quantification of viral load was done by real time quantitative RT-PCR (RT-qPCR), using primers D359 (5′-CAATAGGTGCGAGAGAAGCACAC-3′) and D360 (5′-TAACCCCTGAACGCCCAGTAAG-3′). RT-qPCR reaction mix was prepared using the One-Step SYBR® Prime Script™ RT-PCR Kit II (Takara) with 100 ng RNA per reaction and following the instructions provided by the manufacturer. Amplifications were done using the StepOnePlus™ Sequence Analyzer 7500 (Applied Biosystems) with the following profile: 5 min at 42°C, 10 s at 95°C following 40 cycles of 5 s at 95°C and 34 s at 60°C. RT-qPCR reactions were performed in triplicate for each sample. Quantification results were further examined using SDS 7500 software v.2.2.2 (Applied Biosystems). Standards were prepared by linearization with *Bgl*II of the plasmid containing the TuMV infectious clone, followed by *in vitro* transcription using the mMessage mMachine® SP6 (Ambion) kit and following the manufacturer’s instructions. Known amounts of *in vitro* transcribed RNA were then added to a healthy plant total RNA preparation.

As a normalized measure of the rate of virus accumulation we used the Malthusian growth parameter, *m*, estimated as the slope of the linear regression of the log-transformed values of virus concentration to time (hpi). For statistical analyses, *m* values were fitted to a generalized linear model (GLM) with a Gamma distribution and a log-link function; “host” and “*pipo*” were treated as factorial random factors and “hpi” as a covariable in the model.

### Minimum time necessary to trigger systemic infection

To study differences in the speed of colonization of systemic tissues of both TuMV *pipo* alleles, we followed the protocol described by Lafforgue *et al*. [28]. In short, ground infected tissue of *N. benthamiana*, obtained as described in the previous section, was re-suspended in inoculation buffer and the concentration of viral RNA was made equal for both genotypes. This sap was used to mechanically inoculate 158 four-week old *N. benthamiana,* 220 three-week old *A. thaliana* and 109 four-week old *B. rapa* plants (ca. half of the plants were inoculated with each *pipo* allele). Inoculated plants were maintained in a growth chamber under conditions of 16 h of light at 25°C and 8 h of darkness at 22°C. Inoculated leaves were removed at 24, 48, 72, 96, 120, and 144 hpi, and plants were maintained for up to two weeks under the same growth conditions. The effect of removing the inoculated leaf at different days post-inoculation (dpi) on the probability of systemic infection was determined by ascertaining whether there was GFP fluorescence in non-inoculated leaves after 9 dpi in the case of *N. benthamiana* and after 15 dpi for *A. thaliana* and *B. rapa*. GFP fluorescence was observed as described above. The frequency of systemically infected plants was fitted to a Binomial logistic regression equation using GLM; “host”, “*pipo*” and “dpi” (i.e., time at which inoculated leaves were removed) were treated as factorial random factors in the model.

### Rate of expansion of primary infection foci

Four-week old *N. benthamiana*, three-week old *A. thaliana* and four-week old *B. rapa* plants were inoculated with ground infected tissue from *N. benthamiana* as described above. Inoculated leaves were examined at 48, 72 and 96 hpi with a Leica MZ16F stereomicroscope for formation of primary foci of infection. An individual photo was taken for each of many foci per leaf, and zoom was adjusted to the size of the focus and the intensity of fluorescent signal. All photos were normalized introducing the scale of amplification. The area (mm^2^) of a number of primary infection foci (103 from *N. benthamiana*, 45 from *A. thaliana* and 194 from *B. rapa*; ca. half for each of the two *pipo* alleles) was calculated using a MATLAB script, using the functions in the Image Processing Toolbox (MathWorks, Inc.). For statistical analyses, surfaces were divided by π and root-square transformed into effective radii (mm). The transformed data were fitted to a GLM with a Gamma distribution and log-link function; “host” and “*pipo*” were treated as factorial random factors and “hpi” as covariable in the model.

## Results

### Intra-specific variation in pipo length

Five or less isolates were available for 35 of the analyzed viruses, whereas more than 10 sequences were obtained only for eight viruses (Additional file 1: Table S1). Despite the reduced number of isolates available for most viruses, eight showed intra-specific alternative stop codons involving variable PIPO sizes (Table 1 and Additional file 1: Tables S2-S9). PVY showed the largest number of alternative stop codons (eight) among the 80 isolates included in the study. PPV and *Papaya ringspot virus* (PRSV) (with 69 and nine isolates, respectively) showed three alternative stop codons, whereas *Pea seed-borne mosaic virus* (PSbMV), *Zucchini yellow mosaic virus* (ZYMV), *Sugarcane mosaic virus* (SCMV), *Potato virus A* (PVA), and TuMV (with 3, 12, 13, 7, and 101 isolates, respectively) only presented two alternative stop codons each (Table 1). Whereas the minimal difference in PIPO size of only one amino acid was observed for ZYMV and SCMV, differences of six amino acids were found for PPV, nine amino acids for TuMV, and 11 amino acids for PRSV and PVA. Interestingly, the largest differences in size were observed for PVY (up to 31 amino acids) and especially for PSbMV, with three isolates having PIPO of 79 amino acids long and a fourth isolate having a protein as long as 168 amino acids. With respect to the frequency at which alternative stop codons were present in each virus, clear differences were also observed (Table 1), although it is worth mentioning that these frequencies do not take into account phylogenetic non-independence. A predominant stop codon was present in PPV, ZYMV, PVA, and TuMV. In the rest of viruses, however, alternative stop codons were present at similar frequencies, or at least no particular codon predominated.

**Table 1:** **Viruses showing alternative stop codons in the *pipo *** **ORF**

**GenBank accession**	**Virus**	**PIPO size**	**# Isolates**	**Alternative stop codons**	**Frequency of functional**
NC_001445	*Plum pox virus*	99	69	100 (1)	0.014
				103 (59)	0.87
				106 (68)	0.116
NC_001616	*Potato virus Y*	75	80	62 (1)	0.0125
				65 (1)	0.0125
				73 (21)	0.26
				74 (1)	0.0125
				76 (58)	0.64
				77 (79)	0.05
				93 (80)	0.0125
NC_001671	*Pea seed-borne mosaic virus*	79	3	80 (2)	0.67
				169 (1)	0.33
NC_001785	*Papaya ringspot virus*	72	9	69 (1)	0.11
				73 (8)	0.78
				80 (4)	0.11
NC_003224	*Zucchini yellow mosaic virus*	76	12	76 (1)	0.083
				77 (12)	0.917
NC_003398	*Sugarcane mosaic virus*	80	13	81 (9)	0.69
				82 (8)	0.31
NC_004039	*Potato virus A*	83	7	84 (6)	0.857
				95 (7)	0.143
NC_002509	*Turnip mosaic virus*	60	101	61 (87)	0.861
				70 (87)	0.139

For each virus, the GenBank accession number of the reference sequence and its estimated PIPO size is given. The last three columns indicate the number of available isolates for each virus, the observed alternative stop codons (the number of isolates showing each stop codon is indicated within parenthesis) and the frequency at which a stop codon is the functional one for each virus, respectively. In the last column, frequencies are obtained for each virus by dividing the number of times a given stop codon appears first in the P3N-PIPO sequence by the total number of isolates.

### Evaluating the strength of selection on alternative stop codons

Visual inspection allowed us to see that some changes in PIPO length resulted in nonsynonymous changes at the corresponding position of the P3 protein. This observation prompts the question of whether stop mutations were selected for their effects on P3N-PIPO or on P3. To answer this question, we ran selection analyses to evaluate the strength of selection on *P3* for PPV, PVY and TuMV datasets, since they had the largest number of isolates. For PPV, no codons were found to be under positive selection in *P3*. For PVY, a single positively selected site was detected with FEL and IFEL methods at codon 179 in *P3* (919 for the polyprotein), a site within the *pipo* ORF. A marginally significant positive selection (*P* = 0.052) was detected by FEL at codon 233 on *P3* (amino acid 973 for the polyprotein). This codon is partially coincident with codon 62 from *pipo*, where a stop codon exists in one isolate (Table 1), besides showing a significant excess of non-synonymous changes at the corresponding positions of P3 protein. Taking these results into account, we constructed a contingency table classifying positively selected sites in *P3* according to whether they occur in *pipo* or in the rest of *P3* cistron. An excess of cases of positive selection within *pipo* was detected (*P* = 0.044). For TuMV, three positively selected sites were detected with the FEL method at codons 175, 180 and 203 on *P3* (998, 1003 and 1026 for the polyprotein), all of them occurring in *pipo*. These three codons partially overlap with codons 11–12, 16–17, and 39–40, respectively, from *pipo*. An additional selected site outside *pipo* was also detected with the IFEL method at codon 238 on *P3* (amino acid 1061 for the polyprotein). As previously observed for PVY, an excess of cases of positive selection within *pipo* was detected for TuMV (*P* = 0.035). In any case, the strength of selection on *P3* seems not to account for the presence of alternative stop codons in PPV, PVY and TuMV *pipo* ORF.

### Associations between pipo length and biological factors

Next, we sought for an association between the distribution of stop codons and three biological factors: host species, geographic origin and viral strain. In several cases, this association was obvious after visual inspection of the data. For PPV, the distribution of stop codons was related with the three biological factors (Additional file 1: Table S2). For instance, five out of the six isolates lacking stop codon 103 belonged to strain C while 47 out of the 48 isolates with this stop codon belonged to strain D. Also, it is noticeable that all isolates from cherry belonged to strain C and were European, which is in agreement with previous studies showing an association among host range, viral strain and geographic distribution [29,30]. Regarding PVY, in addition to the previously described association between isolates from strain C and the stop codon 77 [13], strain N isolates predominantly contained stop codon 73, while stop codon 76 was the most abundant among strain O isolates (Additional file 1: Table S3). For TuMV, a clear example of association is that stop codon 61 was present in all Asian isolates, whereas it was absent in some isolates from Europe, America and Oceania (Additional file 1: Table S4). Finally, SCMV stop codon 81 was present in all the maize isolates but absent in the sugarcane isolates (Additional file 1: Table S5).

All these associations were statistically significant (Fisher's exact test, *P* < 0.05 in all cases). However, it is necessary to recall that isolates from a virus do not represent independent observations but are phylogenetically related, thus jeopardizing the results from these simple association tests. Therefore, phylogenetic information has to be taken into account for proper statistical analyses. As before, we focused on PPV, PVY and TuMV data sets, since they encompassed large number of isolates. First, we wanted to test whether phylogenetic structure may explain the association between the distribution of stop codons and the three biological factors. For each virus, independent phylogenetic reconstructions were performed for the analysis of each factor, since the number of isolates for which information was available varied among factor. Phylogenetic analyses with the twelve data sets (four for each virus) were performed as described in the Methods section. After obtaining the MCC phylogenetic tree, BATS [24] was used to investigate the distribution of the three factors and of *pipo* length, along the phylogenies (as described in [13]). For PPV, these analyses showed that the three factors were not randomly distributed over the phylogenies but had a tendency to segregate on different clusters, and the same happened with the length of *pipo* (Table 2). To the extent of our knowledge, these analyses provide the first results concerning population structure and phylodynamics for PPV. For PVY and TuMV, significant associations were also observed (Tables 3 and 4, respectively), confirming previous results [13], although for PVY it was only marginally significant for the host species factor. Regarding TuMV, 15 different species were used for the analysis of the host factor. However, almost half of host species belong to the *Solanaceae* family (Table 4), and thus could not be treated as independent realizations of the trait. To solve this problem, we re-ran BATS but grouped isolates as belonging to the *Brassicaceae* (80 isolates) or the non-*Brassicaceae* (11 isolates). In this case, a significant association was observed, although it only arose because of the non-random distribution across the phylogeny of isolates from the *Brassicaceae* group (*MC* = 32.514, *P* = 0.011).

**Table 2:** **Association between geographic origin, host species, viral strain, and length of *pipo *** **allele with the distribution of viral isolates in the MCC phylogenetic tree obtained for PPV**

**Analyses**	**# Isolates**	**Association statistics**	**Test value**	***P***
Geographic origin		*PS*	22.722	< 0.001
		*AI*	4.167	< 0.001
Asia	39	*MC*	4.205	0.010
America	20	*MC*	2.283	0.010
Europe	6	*MC*	1.120	0.010
Host species		*PS*	24.777	< 0.001
		*AI*	4.614	< 0.001
Apricot	38	*MC*	3.993	0.010
Peach	16	*MC*	1.855	0.010
Plum	6	*MC*	1.144	0.030
Cherry	4	*MC*	1.054	0.010
Almond	1	*MC*	NA^1^	
Strain		*PS*	5.850	< 0.001
		*AI*	1.222	< 0.001
D	53	*MC*	11.381	0.010
C	5	*MC*	1.145	0.010
M	1	*MC*	NA^1^	
PIPO length		*PS*	9.598	< 0.001
		*AI*	2.070	< 0.001
103	59	*MC*	9.409	0.010
106	9	*MC*	1.345	0.010
100	1	*MC*	NA^1^	

*AI*: Association index, *PS*: Parsimony score, *MC*: Maximum monophyletic clade size.

^1^insufficient sample size (*n* < 2).

**Table 3:** **Association between geographic origin, host species, viral strain, and length of *pipo *** **allele with the distribution of viral isolates in the MCC phylogenetic tree obtained for PVY**

**Analyses**	**# Isolates**	**Association statistics**	**Test value**	***P***
Geographic origin		*PS*	36.260	< 0.001
		*AI*	4.928	< 0.001
Asia	17	*MC*	1.739	0.040
Europe	32	*MC*	2.768	0.180
America	27	*MC*	2.354	0.010
Africa	1	*MC*	NA^1^	
Oceania	1	*MC*	NA^1^	
Host species		*PS*	7.922	0.040
		*AI*	1.416	0.060
Potato	63	*MC*	11.566	0.030
Tobacco	4	*MC*	1.068	0.050
Pepper	1	*MC*	NA^1^	
Black nightshade	1	MC	NA^1^	
Wild tomato	1	*MC*	NA^1^	
Tomato	1	*MC*	NA^1^	
Strain		*PS*	27.433	< 0.001
		*AI*	4.092	< 0.001
O	46	*MC*	4.361	0.010
N	23	*MC*	2.148	0.010
C	8	*MC*	1.120	0.010
PIPO length		*PS*	27.323	< 0.001
		*AI*	4.141	< 0.001
76	51	*MC*	4.410	0.010
73	22	*MC*	2.046	0.010
77	4	*MC*	1.063	1
93	1	*MC*	NA^1^	
62	1	*MC*	NA^1^	
74	1	*MC*	NA^1^	
65	1	*MC*	NA^1^	

*AI*: Association index, *PS*: Parsimony score, *MC*: Maximum monophyletic clade size.

^1^insufficient sample size (*n* < 2).

**Table 4:** **Association between geographic origin, host species, viral strain, and length of *pipo *** **allele with the distribution of viral isolates in the MCC phylogenetic tree obtained for TuMV**

**Analyses**	**# Isolates**	**Association statistics**	**Test value**	***P***
Geographic origin		*PS*	30.362	< 0.001
		*AI*	5.394	< 0.001
Asia	67	*MC*	5.698	< 0.001
Europe	28	*MC*	2.298	< 0.001
America	5	*MC*	1.066	< 0.001
Africa	1	*MC*	NA^1^	
Oceania	1	*MC*	NA^1^	
Host species		*PS*	44.461	< 0.001
		*AI*	7.139	< 0.001
*Raphanus sativus*	45	*MC*	3.662	0.090
*Brassica oleraceae*	12	*MC*	1.372	0.200
*Brassica napus*	7	*MC*	1.157	0.020
*Brassica campestris*	5	*MC*	1.084	1
*Brassica pekinensis*	5	*MC*	1.044	0.040
*Armoracia rusticana*	3	*MC*	1.024	1
*Anemone coronaria*	2	*MC*	1.008	1
*Calendula officinalis*	2	*MC*	1.004	1
*Lactuca sativa*	2	*MC*	1	1
*Allium ampeloprasum*	1	*MC*	NA^1^	
*Alliaria officinalis*	1	*MC*	NA^1^	
*Eustoma russellianum*	1	*MC*	NA^1^	
*Ranunculus asiaticus*	1	*MC*	NA^1^	
*Sisymbrium loeselli*	1	*MC*	NA^1^	
*Zaterdeschia spp.*	1	*MC*	NA^1^	
Strain		*PS*	28.165	< 0.001
		*AI*	4.894	< 0.001
BR	28	*MC*	4.855	0.010
B	29	*MC*	2.522	0.010
(B)	2	*MC*	2.522	1
B(R)	1	*MC*	NA^1^	
PIPO length		*PS*	13.392	< 0.001
		*AI*	2.598	< 0.001
61	87	*MC*	11.401	0.010
70	87	*MC*	1.475	0.010

*PS*: Parsimony score, *AI*: Association index, *MC*: Maximum monophyletic clade size.

^1^insufficient sample size (*n* < 2).

Next we used Felsenstein’s independent contrasts method [31] to evaluate the association between the distribution of stop codons and each of the three biological factors for these three viruses while continuing to take the underlying phylogenies into consideration. No significant correlations were observed for any of the viruses (correlation coefficients ranging between -0.050 and 0.120, and *P* > 0.33 in all cases).

### Pipo length affects different fitness components of TuMV

As discussed in the previous section, Tables 2, 3 and 4 show that the phylogenetic distribution of PPV, PVY and TuMV isolates can be explained by the host species as well as by the length of *pipo*, suggesting that the apparent association between both traits may merely result from the phylogenetic relationships between isolates. To experimentally test whether *pipo* length had an effect on virus infection in different host species, we evaluated four different fitness components for both TuMV strains carrying the two *pipo* alleles of different lengths found for this virus (Table 1), *pipo*^*61*^ and *pipo*^*70*^, across three alternative host species belonging to two botanical families, the *Solanaceae* (*N. benthamiana*) and the *Brassicaceae* (*B. rapa* and *A. thaliana*).

The first fitness component analyzed was infectivity, that is, the probability of establishing an infection upon inoculation of a given viral dose [32,33]. Consistent with the role of P3N-PIPO as viral movement protein [8,9], no differences in infectivity were observed between the two alleles (data not shown).

**Figure 1:**
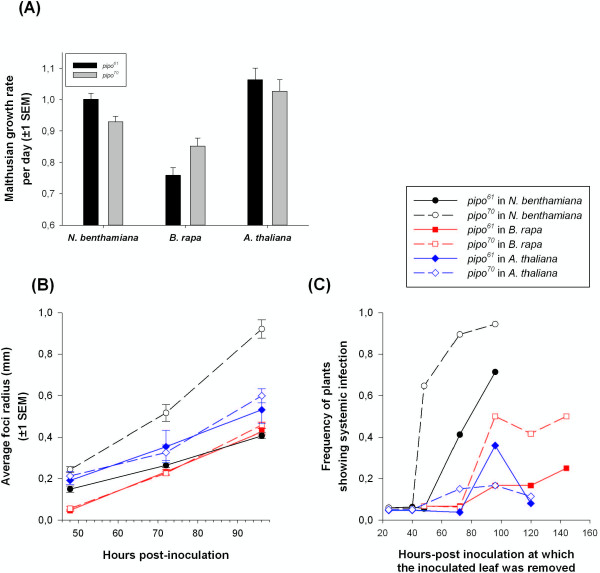
**Effects of two *pipo *alleles differing in length on TuMV biological properties. (A)** Differences in accumulation rate between the wild-type *pipo*^61^ and the long *pipo*^70^ alleles across three experimental host species. Accumulation rate was measured as the per-day Malthusian growth parameter. **(B)** Rates of expansion of infection foci for the two alleles in the three experimental host species. **(C)** Speed of systemic infection for the two alleles in the three experimental host species. The time necessary to produce a systemic infection was measured as described in Lafforgue *et al*. [28]. Panels **(B)** and **(C)** share the same legend: solid symbols and lines correspond to the *pipo*^*61*^ allele, whereas open symbols and dashed lines to the *pipo*^*70*^ allele.

The second fitness component evaluated was Malthusian growth rate, which is a proxy for the rate of virus accumulation per day (Figure 1A). Significant differences in Malthusian growth rate exist between the three hosts (GLM test of “host” effect: *χ*^2^ = 70.8398, 2 d.f., *P* < 0.001), with growth rates being larger in *A. thaliana* and *N. benthamiana* and lower in *B. rapa*. More interestingly, a highly significant host-species-dependent effect of the *pipo* allele was found (GLM test of “host × *pipo*” interaction: *χ*^2^ = 13.994, 2 d.f., *P* = 0.001). TuMV-*pipo*^*61*^ grew 7.8% faster in *N. benthamiana* and 3.6% faster in *A. thaliana* than the virus carrying the longer *pipo*^*70*^ allele. In contrast, the virus carrying the *pipo*^*70*^ allele grew 10.9% faster in *B. rapa*.

Next, we tested for differences between alleles in the rate of cell-to-cell movement by quantifying the average effective radius of expanding foci at increasing hpi, and comparing these dynamics across hosts (Figure 1B). Overall, no effect of the *pipo* alleles on the rate of foci expansion was detected (i.e., the slope of the regression lines; GLM test of “*pipo* × hpi” interaction: *χ*^2^ = 3.559, 2 d.f., *P* = 0.169). However, significant differences among slopes exist in the case of *N. benthamiana* plants (*χ*^2^ = 8.604, 1 d.f., *P* = 0.003), with TuMV-*pipo*^*61*^ having a 2.55 (ratio of slopes) times faster cell-to-cell expansion than the virus carrying the long allele. No significant effects were observed in the other two hosts.

Finally, the fourth viral fitness component studied was the time required for the virus to exit the inoculated leaf and initiate a systemic infection. The faster a virus expands by cell-to-cell movement, the shorter the time necessary for the virus to reach the phloem and move upwards in the plant, following photo-assimilates towards sink tissues. Thus, in a set of plants this will translate into a higher frequency of systemic infections. To evaluate this, we followed the experimental protocol described by Lafforgue *et al*. [28], which consists of inoculating plants, removing the inoculated leaf after several hpi, and counting the number of plants developing systemic infection in each case. Figure 1C shows these data. As in the case of the cell-to-cell movement rate, no overall effect of the *pipo* allele was found (GLM test of the “*pipo*” main factor: *χ*^2^ = 0.003, 1 d.f., *P* = 0.956), although there was a significant three-way interaction (GLM test of the “host × *pipo* × hpi”: *χ*^2^ = 12.669, 2 d.f., *P* = 0.002). This interaction demonstrates that the time required for triggering a systemic infection indeed depends on the interaction between the host species and the *pipo* allele. At one extreme, systemic infection occurs sooner in *N. benthamiana* than in the other two hosts, and it is faster for the *pipo*^*70*^ allele (Figure 1C). At the other extreme, time to establishing a systemic infection is longer in *A. thaliana*, with no significant differences among *pipo* alleles (Figure 1C). The case of *B. rapa* is intermediate among these two extremes, again with the longer allele promoting earlier systemic movement than the shorter one (Figure 1C).

Taken together, these results confirm that viral fitness depends on the interaction between the *pipo* allele and the host species being infected, thus providing an adaptive explanation for the different prevalence of *pipo* alleles among host species.

## Discussion

The fact that *pipo* is embedded within *P3* imposes strong functional and sequence constrains to *Potyvirus* evolution. The *P3* cistron has been shown to be involved in adaptation to new hosts or overcoming resistance or tolerance in many potyviruses. This effect has been observed for *Tobacco etch virus*[34], TuMV [35-37], PSbMV [38], ZYMV [39,40], and *Soybean mosaic virus* (SMV) [41]. However, most of the virulence determinants described fall outside the P3N-PIPO protein. Interestingly, a key virulence determinant of SMV overlaps with both *P3* and *pipo* ORFs, although it has been shown that these effects on virulence are due to the amino acid change in P3 but not in P3N-PIPO [42]. Also, a single mutation in the *P3* cistron of TuMV that is essential for adaptation to *Raphanus sativus* also changes an amino acid in the P3N-PIPO protein [43], but the role of P3N-PIPO in this adaptation event remains to be determined. In any case, the fact that *pipo* is expressed as a fusion product with the N-terminal portion of *P3* does not preclude from the potential influence of P3N-PIPO on virulence determinants mapped onto *P3* and upstream P3N-PIPO. Since P3N-PIPO can also be involved in other functions [11], these potential multifunctional properties must be considered when analyzing particular positions in this protein.

Our results suggest that intra-specific variability in *pipo* size is a common feature in potyviruses. In this sense, five out of the eight viruses showing more than 10 isolates presented alternative stop codons, whereas this feature was only observed for three out of the 40 viruses for which less than 10 isolates were available. To date, experiments with the *pipo* ORF have consisted of introducing nonsynonymous mutations [44] or drastically premature stop codons [7,9]. However, experimental evidence of the potential effects of small differences in P3N-PIPO length, apart from those showed in the present work, is still missing. Consequently, further studies will be needed to understand whether the intra-specific variation in stop codon usage consists of neutral or nearly neutral polymorphisms that have been incorporated by drift events, or whether it plays a significant adaptive role in terms of host-range expansion, geographical dispersion and/or strain diversification.

Our experimental results confirm variability of the adaptive value of different *pipo* stop codons. We have not observed differences in infectivity, whereas we did observe differences in spread and accumulation. These observations suggest that the length of different alleles has no effect on initiating the infection. On the other hand, *pipo* length does have quantitative effects on virus spread, as might be expected for a mutation affecting the viral movement protein. Moreover, these effects are host dependent, thus further enhancing their potential selective value: certain alleles may be fitter in some hosts while other alleles may be so in alternative hosts.

## Conclusions

Available information for the different poytivirus species suggests that intra-specific variability in *pipo* length is common. Our results also indicate that this variation may be maintained by host-driven selection, although further studies using other potyvirus models will be needed to shed some light on the generality of the results here shown.

## Competing interests

The authors declared that they have no competing interests.

## Authors’ contributions

JH and JMC performed the computational analyses, JH performed the experiments, SFE analyzed the empirical data, JMC and SFE conceived the study, JMC and SFE drafted the manuscript. All authors have read and approved the final manuscript.

## Supplementary Material

Additional file 1: Table S1 Potyviruses analyzed in the present study. For each virus, the GenBank accession number of the reference sequence and its estimated PIPO size is given. The last two columns indicate the number of available isolates for each virus and the observed alternative stop codons, if present. The number of isolates showing a given stop codon is indicated in parentheses. **Table S2**. Available information for isolates belonging to *Plum pox virus*. The last three columns indicate the presence (+) or absence (-) of the alternative stop codons in each isolate. **Table S3**. Available information for isolates belonging to *Potato virus Y*. The last seven columns indicate the presence (+) or absence (-) of the alternative stop codons in each isolate. **Table S4**. Available information for isolates belonging to *Turnip mosaic virus*. The last two columns indicate the presence (+) or absence (-) of the alternative stop codons in each isolate. **Table S5**. Available information for isolates belonging to *Sugarcane mosaic virus*. The last two columns indicate the presence (+) or absence (-) of the alternative stop codons in each isolate. **Table S6**. Available information for isolates belonging to *Pea seed-borne mosaic virus*. The last two columns indicate the presence (+) or absence (-) of the alternative stop codons in each isolate. **Table S7**. Available information for isolates belonging to *Papaya ringspot virus*. The last three columns indicate the presence (+) or absence (-) of the alternative stop codons in each isolate. **Table S8**. Available information for isolates belonging to *Zucchini yellow mosaic virus*. Strain is not available for any of the isolates. The last two columns indicate the presence (+) or absence (-) of the alternative stop codons in each isolate. **Table S9.** Available information for isolates belonging to *Potato virus A*. The last two columns indicate the presence (+) or absence (-) of the alternative stop codons in each isolate. Click here for file
