# Supplementary material for: Intra-specific variability and biological relevance of P3N-PIPO protein length in potyviruses
Source: BMC Evol Biol. 2013 Nov 13;13:249. doi: 10.1186/1471-2148-13-249 (PMC3840659; doi:10.1186/1471-2148-13-249)
Supplement: Additional file 1: Table S1 — Potyviruses analyzed in the present study. For each virus, the GenBank accession number of the reference sequence and its estimated PIPO size is given. The last two columns indicate the number of available isolates for each virus and the observed alternative stop codons, if present. The number of isolates showing a given stop codon is indicated in parentheses. Table S2. Available information for isolates belonging to Plum pox virus. The last three columns indicate the presence (+) or absence (-) of the alternative stop codons in each isolate. Table S3. Available information for isolates belonging to Potato virus Y. The last seven columns indicate the presence (+) or absence (-) of the alternative stop codons in each isolate. Table S4. Available information for isolates belonging to Turnip mosaic virus. The last two columns indicate the presence (+) or absence (-) of the alternative stop codons in each isolate. Table S5. Available information for isolates belonging to Sugarcane mosaic virus. The last two columns indicate the presence (+) or absence (-) of the alternative stop codons in each isolate. Table S6. Available information for isolates belonging to Pea seed-borne mosaic virus. The last two columns indicate the presence (+) or absence (-) of the alternative stop codons in each isolate. Table S7. Available information for isolates belonging to Papaya ringspot virus. The last three columns indicate the presence (+) or absence (-) of the alternative stop codons in each isolate. Table S8. Available information for isolates belonging to Zucchini yellow mosaic virus. Strain is not available for any of the isolates. The last two columns indicate the presence (+) or absence (-) of the alternative stop codons in each isolate. Table S9. Available information for isolates belonging to Potato virus A. The last two columns indicate the presence (+) or absence (-) of the alternative stop codons in each isolate. [file 1471-2148-13-249-S1.pdf]

Table S1. Potyviruses analyzed in the present study. For each virus, the GenBank accession number of the reference sequence and its estimated PIPO size is given. The last two columns indicate the number of available isolates for each virus and the observed alternative stop codons, if present. The number of isolates showing a given stop codon is indicated in parentheses.

GenBank accession	Virus	Size	# Isolates	Stop codons
NC_000947	*Japanese yam mosaic virus*	60	1	61
NC_001445	*Plum pox virus*	99	69	100 (1), 103 (59), 106 (68)
NC_001517	*Pepper mottle virus*	75	18	76
NC_001555	*Tobacco etch virus*	99	3	100
NC_001616	*Potato virus Y*	75	80	62 (1), 65 (1), 73 (21), 74 (1), 76 (58), 77 (79), 93 (81)
NC_001671	*Pea seed-borne mosaic virus*	79	3	80 (3), 169 (1)
NC_001768	*Tobacco vein mottling virus*	91	2	92
NC_001785	*Papaya ringspot virus*	72	9	69 (1), 73 (8), 80 (4)
NC_001841	*Sweet potato feathery mottle virus*	67	3	68
NC_002509	*Turnip mosaic virus*	60	101	61 (87), 70 (87)
NC_002600	*Peanut mottle virus*	75	1	76
NC_002634	*Soybean mosaic virus*	75	64	76
NC_003606	*Johnsongrass mosaic virus*	91	1	92
NC_003224	*Zucchini yellow mosaic virus*	76	12	76 (1), 77 (12)
NC_003377	*Maize dwarf mosaic virus*	80	4	81
NC_003397	*Bean common mosaic virus*	75	5	76
NC_003398	*Sugarcane mosaic virus*	80	13	81 (10), 82 (8)
NC_003399	*Scallion mosaic virus*	73	1	74
NC_003492	*Bean yellow mosaic virus*	79	10	80
NC_003536	*Clover yellow vein virus*	60	1	61
NC_003537	*Dasheen mosaic virus*	94	1	95
NC_003605	*Lettuce mosaic virus*	75	5	76
NC_003742	*Cocksfoot streak virus*	83	2	84

NC_004010	*Potato virus V*	69	1	70
NC_004011	*Leek yellow stripe virus*	74	4	75
NC_004013	*Cowpea aphid-borne mosaic virus*	73	3	74
NC_004035	*Sorghum mosaic virus*	80	3	81
NC_004039	*Potato virus A*	83	7	84 (6), 95 (7)
NC_004047	*Bean common mosaic necrosis virus*	73	6	74
NC_004426	*Wild potato mosaic virus*	69	1	70
NC_004573	*Peru tomato mosaic virus*	71	4	72
NC_004752	*Yam mosaic virus*	61	1	62
NC_005028	*Papaya leaf-distortion mosaic virus*	75	2	76
NC_005029	*Onion yellow dwarf virus*	75	5	76
NC_005288	*Lily mottle virus*	62	3	63
NC_005304	*Beet mosaic virus*	115	3	116
NC_005778	*Chilli veinal mottle virus*	72	6	73
NC_006262	*Watermelon mosaic virus*	75	28	76
NC_007147	*Pennisetum mosaic virus*	81	2	82
NC_007180	*Thunberg fritillary virus*	80	2	81
NC_007216	*Wisteria vein mosaic virus*	75	1	76
NC_007433	*Shallot yellow stripe virus*	79	2	80
NC_007728	*East Asian Passiflora virus*	75	2	76
NC_007913	*Konjac mosaic virus*	81	1	82
NC_008028	*Daphne mosaic virus*	72	2	73
NC_008393	*Pepper severe mosaic virus*	74	1	75
NC_008558	*Blackberry virus Y*	110	1	111
NC_008824	*Narcissus degeneration virus*	76	1	77

Table S2. Available information for isolates belonging to *Plum pox virus*. The last three columns indicate the presence (+) or absence (-) of the alternative stop codons in each isolate.

GenBank accession	Host	Continent	Strain	100	103	106
HQ840523	cherry	Europe	C	-	-	+
HQ840522	cherry	Europe	C	-	-	+
HQ840521	cherry	Europe	C	-	-	+
AY912055	plum	America		-	-	+
EU734794	apricot	Asia		-	-	+
AY677114	apricot	Asia		-	-	+
FM955843		Europe	M	-	-	+
HQ840517	cherry	Europe	C	-	-	+
Y09851	cherry	Europe	C	-	-	+
AB576080	apricot	Asia	D	-	+	+
AB576079	apricot	Asia	D	-	+	+
AB576078	apricot	Asia	D	-	+	+
AB576077	apricot	Asia	D	-	+	+
AB576076	apricot	Asia	D	-	+	+
AB576075	apricot	Asia	D	-	+	+
AB576074	apricot	Asia	D	-	+	+
AB576073	apricot	Asia	D	-	+	+
AB576072	apricot	Asia	D	-	+	+
AB576071	apricot	Asia	D	-	+	+
AB576070	apricot	Asia	D	-	+	+
AB576069	apricot	Asia	D	-	+	+
AB576068	apricot	Asia	D	-	+	+
AB576067	apricot	Asia	D	-	+	+
AB576066	apricot	Asia	D	-	+	+
AB576065	apricot	Asia	D	-	+	+

AB576064	apricot	Asia	D	-	+	+
AB576063	apricot	Asia	D	-	+	+
AB576062	apricot	Asia	D	-	+	+
AB576061	apricot	Asia	D	-	+	+
AB576060	apricot	Asia	D	-	+	+
AB576059	apricot	Asia	D	-	+	+
AB576058	apricot	Asia	D	-	+	+
AB576057	apricot	Asia	D	-	+	+
AB576056	apricot	Asia	D	-	+	+
AB576055	apricot	Asia	D	-	+	+
AB576054	apricot	Asia	D	-	+	+
AB576053	apricot	Asia	D	-	+	+
AB576052	plum	Asia	D	-	+	+
AB576051	apricot	Asia	D	-	+	+
AB576050	apricot	Asia	D	-	+	+
AB576049	apricot	Asia	D	-	+	+
AB576048	apricot	Asia	D	-	+	+
AB576047	apricot	Asia	D	-	+	+
AB576046	apricot	Asia	D	-	+	+
AB576045	apricot	Asia	D	-	+	+
AB545926	apricot	Asia	D	-	+	+
EU117116	plum	Europe		-	+	+
EF640939	peach	America		-	+	+
EF640938	peach	America		-	+	+
EF640937	peach	America		-	+	+
EF640936	peach	America	D	-	+	+
EF640935	peach	America	D	-	+	+
EF640934	peach	America	D	-	+	-

EF640933	peach	America	D	-	+	+
AM933761	plum	Europe		-	+	+
DQ465243	peach	America	D	-	+	+
DQ465242	peach	America	D	-	+	+
AY953267	peach	America	D	-	+	+
AY953266	plum	America	D	-	+	+
AY953265	almond	America	D	-	+	+
AY953264	peach	America	D	-	+	+
AY953263	peach	America	D	-	+	+
AY953262	peach	America	D	-	+	+
AY953261	plum	America	D	-	+	+
AY912058	peach	America	D	-	+	+
AY912057	peach	America	D	-	+	+
AY912056	peach	America	D	-	+	+
NC_001445				+	-	+
X81083	cherry		C	-	+	+

Table S3. Available information for isolates belonging to *Potato virus Y*. The last seven columns indicate the presence (+) or absence (-) of the alternative stop codons in each isolate.

GenBank accession	Host	Continent	Strain	62	65	73	74	76	77	93
FJ666337	potato	Europe	N	-	-	-	-	+	+	+
AB185833	potato	Asia	O	-	-	-	-	+	+	+
EF026076	potato	America	O	-	-	-	-	+	+	+
EF026075	potato	America	O	-	-	-	-	+	+	+
AY884985	potato	America	O	-	-	-	-	+	+	+
AY884982	potato	America	O	-	-	-	-	+	+	+
FJ643478	potato	America	O	-	-	-	-	+	+	+
FJ643477	potato	America	O	-	-	-	-	+	+	+
AB461453	potato	Asia	O	-	-	-	-	+	+	+
AB461452	potato	Asia	O	-	-	-	-	+	+	+
AB461451	potato	Asia	O	-	-	-	-	+	+	+
GQ200836	potato	Asia	O	-	-	-	-	+	+	+
DQ157179	potato	America	O	-	-	-	-	+	+	+
DQ157178	potato	America	O	-	-	-	-	+	+	+
DQ008213	potato	America	O	-	-	-	-	+	+	+
AY745492	potato	America	O	-	-	-	-	+	+	+
AY745491	potato	America	O	-	-	-	-	+	+	+
AF522296	potato	Africa	N	-	-	-	-	+	+	+
AF463399	tobacco	America	C	-	-	-	-	+	+	+
U09509	potato	America	O	-	-	-	-	+	+	+
M95491	potato	Europe	O	-	-	-	-	+	+	+
EU482153	tomato	Europe	C	-	-	-	-	+	+	+
FJ204166	potato	America	O	-	-	-	-	+	+	+
FJ204165	potato	America	O	-	-	-	-	+	+	+

NC_001616		Europe	N	-	-	-	-	+	+	+
EU182576	tobacco	Asia	N	-	-	-	-	+	+	+
D00441		Europe	N	-	-	-	-	+	+	+
EF558545	potato	Europe	O	-	-	-	-	+	+	+
AJ890350	potato	Europe	O	-	-	-	-	+	+	+
AJ890349	potato	Europe	O	-	-	-	-	+	+	+
AJ890347	potato	Europe	O	-	-	-	-	+	+	+
AJ890345	potato	Europe	O	-	-	-	-	+	+	+
AJ890344	potato	Europe	O	-	-	-	-	+	+	+
AJ890343	tobacco	Europe	O	-	-	-	-	+	+	+
AJ890342	potato	Europe	O	-	-	-	-	+	+	+
AJ889868	potato	Europe	O	-	-	-	-	+	+	+
AJ889867	potato	Europe	O	-	-	-	-	+	+	+
AJ889866	potato	Europe	O	-	-	-	-	+	+	+
EF016294	potato	Europe	O	-	-	-	-	+	+	+
CS434577			O	-	-	-	-	+	+	+
DQ309028	tobacco	America	C	-	-	-	-	+	+	+
AJ585342	potato	Europe	O	-	-	-	-	+	+	+
AJ585196	potato	Europe	O	-	-	-	-	+	+	+
AJ585195	potato	Europe	O	-	-	-	-	+	+	+
A08776				-	-	-	-	+	+	+
AB461450		Asia	O	-	-	-	-	+	+	+
AB461454		Asia	O	-	-	-	-	+	+	+
HM367076	potato	America	O	-	-	-	-	+	+	+
HQ407482	potato	Asia		-	-	-	-	+	+	+
HQ631374	potato	Asia	N	-	-	-	-	+	+	+
JN034046	potato	Asia		-	-	-	-	+	+	+
AY166867	potato	America	N	-	-	+	-	-	+	+

AB270705	potato	Asia	N	-	-	+	-	+	+	+
AY166866	potato	America	N	-	-	+	-	-	+	+
EF026074	potato	America	O	-	-	+	-	+	+	+
AY884984	potato	America	N	-	-	+	-	-	+	+
AY884983	potato	America	N	-	-	+	-	-	+	+
FJ643479	potato	America	O	-	-	+	-	-	+	+
DQ157180	potato	America	N	-	-	+	-	-	-	+
AB331519	potato	Asia	N	-	-	+	-	-	+	+
AB331518	potato	Asia	N	-	-	+	-	+	+	+
AB331517	potato	Asia	N	-	-	+	-	-	+	+
AB331516	potato	Asia	N	-	-	+	-	-	+	+
AB331515	potato	Asia	N	-	-	+	-	-	+	+
AM268435	potato	Oceania	N	-	-	+	-	-	+	+
AJ890346	potato	Europe	O	-	-	+	-	+	+	+
X97895	potato	Europe	N	-	-	+	-	-	+	+
AJ439544	Black nightshade	Europe	C	-	-	+	-	-	+	+
AJ585198	potato	Europe	O	-	-	+	-	-	+	+
AJ585197	potato	Europe	N	-	-	+	-	-	+	+
HM367075	potato	America	O	-	-	+	-	+	+	+
AJ584851	potato	Europe	N	+	-	-	-	+	+	+
AM113988	potato	Europe	O	-	+	-	-	+	+	+
AJ890348	potato	Europe	C	-	-	-	+	+	+	+
AJ439545	wild tomato	Europe	C	-	-	-	-	-	+	+
EU563512	potato	Europe	C	-	-	-	-	-	+	+
FJ214726	ají	America		-	-	-	-	-	+	+
FJ204164	potato	America	O	-	-	-	-	-	+	+
AF237963	pepper	Europe	C	-	-	-	-	-	-	+

CS434575	N	-	-	+	-	-	+	+

Table S4. Available information for isolates belonging to *Turnip mosaic virus*. The last two columns indicate the presence (+) or absence (-) of the alternative stop codons in each isolate.

GenBank accession	Host	Continent	Strain	61	70
AB093604	*Brassica napus*	Europe	B	-	+
AB093606	*Armoracia rusticana*	Europe	B	-	+
AB093607	*B. napus*	Europe	B	-	+
AB093608	*Brassica oleracea*	Europe	B	-	+
AB093610	*B. napus*	America	B	-	+
AB093611	*B. oleracea*	America	B	-	+
AB093612	*Brassica pekinensis*	Oceania	B	-	+
AB252107	*Brassica rapa*	Europe	B	-	+
AB252108	*B. napus*	Europe	B	-	+
AY227024	*B. napus*	America		-	+
D10927	*B. napus*	America		-	+
DQ648591	*Cochlearia armoracia*	Europe		-	+
EF374098	*C. armoracia*	Europe		-	+
HQ637383		Europe		-	+
AB093596	*Limonium sinuatum*	Europe	B	+	+
AB093600	*Raphanus sativus*	Europe	BR	+	+
AB093605	*B. oleracea*	Africa	B	+	+
AB093609	*B. oleracea*	America	B	+	+
AB093613	*R. sativus*	Asia	BR	+	+
AB093614	*Calendula officinalis*	Asia	BR	+	+
AB093617	*R. sativus*	Asia	BR	+	+
AB093618	*R. sativus*	Asia	BR	+	+
AB093619	*R. sativus*	Asia	BR	+	+
AB093620	*R. sativus*	Asia	BR	+	+
AB093621	*R. sativus*	Asia	BR	+	+

AB093622	*B. pekinensis*	Asia	BR	+	+
AB093623	*R. sativus*	Asia	BR	+	+
AB093624	*B. pekinensis*	Asia	BR	+	+
AB093625	*Brassica campestris*	Asia	B	+	+
AB093626	*Brassica spp.*	Asia	BR	+	+
AB093627	*R. sativus*	Asia	BR	+	+
AB105134	*B. oleracea*	Asia	B	+	+
AB105135	*R. sativus*	Asia	BR	+	+
AB252094	*R. sativus*	Asia	BR	+	+
AB252095	*R. sativus*	Asia	BR	+	+
AB252096	*R. sativus*	Asia	BR	+	+
AB252097	*R. sativus*	Asia	BR	+	+
AB252098	*R. sativus*	Asia	BR	+	+
AB252099	*R. sativus*	Asia	BR	+	+
AB252100	*R. sativus*	Asia	BR	+	+
AB252101	*B. pekinensis*	Asia	BR	+	+
AB252102	*Eustoma russellianum*	Asia	BR	+	+
AB252103	*R. sativus*	Asia	BR	+	+
AB252104	*R. sativus*	Asia	BR	+	+
AB252105	*R. sativus*	Asia	BR	+	+
AB252106	*B. campestris*	Asia	B(R)	+	+
AB252109	*R. sativus*	Asia	BR	+	+
AB252110	*R. sativus*	Asia	BR	+	+
AB252111	*B. pakinensis*	Asia	BR	+	+
AB252112	*B. oleracea*	Europe	B	+	+
AB252113	*B. oleracea*	Europe	B	+	+
AB252114	*B. oleracea*	Europe	B	+	+
AB252115	*R. sativus*	Asia	BR	+	+

AB252116	*B. oleracea*	Europe	B	+	+
AB252118	*R. sativus*	Asia	BR	+	+
AB252119	*Brassica spp.*	Asia	B	+	+
AB252120	*R. sativus*	Asia	BR	+	+
AB252121	*R. sativus*	Asia	BR	+	+
AB252123	*R. sativus*	Asia	BR	+	+
AB252124	*B. oleracea*	Asia	B	+	+
AB252126	*R. sativus*	Asia	BR	+	+
AB252127	*R. sativus*	Asia	BR	+	+
AB252128	*R. sativus*	Asia	BR	+	+
AB252129	*R. sativus*	Asia	BR	+	+
AB252130	*R. sativus*	Asia	BR	+	+
AB252131	*R. sativus*	Asia	BR	+	+
AB252132	*R. sativus*	Asia	BR	+	+
AB252133	*B. oleracea*	Europe	B	+	+
AB252134	*R. sativus*	Asia	BR	+	+
AB252135	*Ranunculus asiaticus*	Europe	B	+	+
AB252136	*B. rapa*	Asia	BR	+	+
AB252137	*R. sativus*	Asia	BR	+	+
AB252138	*R. sativus*	Asia	BR	+	+
AB252139	*R. sativus*	Asia	BR	+	+
AB252140	*R. sativus*	Asia	BR	+	+
AB252141	*Lactuca sativa*	Asia	BR	+	+
AB252143	*R. sativus*	Asia	BR	+	+
AB362512	*B. olerasea*		B	+	+
AB440238	*Raphanus rugosum*	Asia		+	+
AB440239	*Sisymbrium loeselii*	Asia		+	+
AF394601		Asia		+	+

AF394602		Asia		+	+
AY090660		Asia	B	+	+
DQ648592	*C. armoracia*	Europe		+	+
EU734434		Asia		+	+
NC_002509	*B. napus*	Europe		+	+
AF530055	*Zantedeschia spp.*	Asia	BR	+	+
AB093597	*Anemone coronaria*	Europe	B	+	-
AB093598	*Alliaria officinalis*	Europe	(B)	+	-
AB093599	*A. coronaria*	Europe	(B)	+	-
AB093601	*C. officinalis*	Europe	BR	+	-
AB093602	*Allium ampeloprasum*	Europe	B	+	-
AB093603	*L. sativa*	Europe	BR	+	-
AB093615	*R. sativus*	Asia	BR	+	-
AB093616	*R. sativus*	Asia	BR	+	-
AB252117	*Allium spp.*	Europe	B	+	-
AB252122	*Brassica spp.*	Europe	B	+	-
AB252125	*B. rapa*	Asia	BR	+	-
AB252142	*R. sativus*	Asia	BR	+	-
AB362513	*R. sativus*	Asia	BR	+	-
EU734433		Asia		+	-

Table S5. Available information for isolates belonging to *Sugarcane mosaic virus*. The last two columns indicate the presence (+) or absence (-) of the alternative stop codons in each isolate.

**GenBank accession**	**Host**	**Continent**	**Strain**	**81**	**82**
JN021933	maize	Asia		+	+
AF494510		Asia		+	+
EU091075	maize	America		+	-
AY569692	maize	Asia		+	-
GU474635	maize	America		+	-
AY149118	maize	Asia		+	+
NC_003398	maize	Asia		+	-
AM110759	maize	Europe		+	-
AJ310105	maize	Asia		+	+
AJ278405		Oceania	A	-	+
AJ310104	sugarcane	Asia		-	+
AJ310103	sugarcane	Asia		-	+
AJ310102	sugarcane	Asia		-	+

Table S6. Available information for isolates belonging to *Pea seed-borne mosaic virus*. The last two columns indicate the presence (+) or absence (-) of the alternative stop codons in each isolate.

GenBank accession	Host	Continent	Strain	80	169
X89997	pea	America	P-4	-	+
NC_001671	pea	Europe	DPD1	+	-
AJ252242	lentil	Europe	P-2	+	-

Table S7. Available information for isolates belonging to *Papaya ringspot virus*. The last three columns indicate the presence (+) or absence (-) of the alternative stop codons in each isolate.

GenBank accession	Host	Continent	Strain	69	73	80
HQ424465	papaya	Asia	P	-	+	-
AY162218	papaya	Asia	P	-	+	-
NC_001785				-	+	+
EU882728	papaya	Asia	P	-	+	-
DQ374153	zucchini	America	W	-	+	+
EF017707	papaya	Asia	P	-	+	+
X96537	papaya	Asia	SM	-	+	-
AY010722	papaya	Asia	W	+	+	-
DQ374152	zucchini	America	W	-	-	+

Table S8. Available information for isolates belonging to *Zucchini yellow mosaic virus*. Strain is not available for any of the isolates. The last two columns indicate the presence (+) or absence (-) of the alternative stop codons in each isolate.

**GenBank accession**	**Host**	**Continent**	**76**	**77**
AM422386	begonia	Asia	-	+
L29569	bitter gourd	Africa	+	-
AY279000	pumpkin	Asia	-	+
AY278999	pumpkin	Asia	-	+
AY278998	pumpkin	Asia	-	+
NC_003224	sponge gourd	Asia	-	+
AB188116	cucumber	Asia	-	+
AB188115	cucumber	Asia	-	+
AJ316229	winter gourd	Asia	-	+
AJ316228	sponge gourd	Asia	-	+
AJ307036	cucumber	Asia	-	+
AJ515911	watermelon	Asia	-	+

Table S9. Available information for isolates belonging to *Potato virus A*. The last two columns indicate the presence (+) or absence (-) of the alternative stop codons in each isolate.

GenBank accession	Host	Continent	Strain	84	95
GU144321	potato	Europe	2	+	+
Z21670				+	+
NC_004039	potato	Europe	3	+	+
AJ131403	tamarillo	Oceania	3	-	+
AJ131402	potato	America	1	+	+
AJ131401	potato	Europe	2	+	+
AJ131400	potato	Europe	1	+	+
